# Anti-Pentraxin Antibodies in Autoimmune Diseases: Bystanders or Pathophysiological Actors?

**DOI:** 10.3389/fimmu.2020.626343

**Published:** 2021-02-16

**Authors:** Benoit Brilland, Emeline Vinatier, Jean-François Subra, Pascale Jeannin, Jean-François Augusto, Yves Delneste

**Affiliations:** ^1^ CHU Angers, Service de Néphrologie-Dialyse-Transplantation, Angers, France; ^2^ Université d’Angers, INSERM, CRCINA, Angers, France; ^3^ CHU Angers, Service d’Immunologie et Allergologie, Angers, France

**Keywords:** pentraxins, autoimmunity, systemic lupus erythematosus, ANCA-associated vasculitis, anti-pentraxin autoantibodies

## Abstract

Pentraxins are soluble innate immunity receptors involved in sensing danger molecules. They are classified as short (CRP, SAP) and long pentraxin subfamilies, including the prototypic long pentraxin PTX3. Pentraxins act mainly as bridging molecules favoring the clearance of microbes and dead cells. They are also involved in many other biological processes, such as regulation of complement activation, inflammation and tissue homeostasis. Autoantibodies directed against pentraxins have been reported in various autoimmune diseases, especially in systemic lupus erythematosus and ANCA-associated vasculitis. In this review, we review the main biological characteristics and functions of pentraxins and summarize data concerning autoantibodies directed against pentraxins in the context of autoimmune diseases and discuss their potential pathological role.

## Introduction

The innate immune system is involved in many physiological processes, including antimicrobial defence, inflammation, initiation and regulation of adaptive immunity and maintenance of tissue homeostasis (1). This system has a cellular arm, composed principally of myeloid cells and innate lymphoid cells, and a humoral arm, including soluble innate immunity receptors, also known as soluble pattern recognition molecules (sPRM) (2). The innate immune system specializes in sensing of so-called “danger signals” that originate from (i) non-self, such as microbes; the microbial motifs recognized by innate cells are called pathogen-associated molecular patterns (PAMPs) or (ii) from modified (or altered) self, mainly dying cells and molecules expressed by damaged cells/tissues or accidentally released in the extracellular environment; these motifs are called damage-associated molecular patterns (DAMPs).

The detection of danger signals by innate immunity receptors is crucial in initiating appropriate immune responses that are fine-tuned to the motifs encountered (tolerance to self, protection against non-self). These receptors are highly conserved molecules called Pattern Recognition Receptors (PRR). PRR recognize a wide variety of ligands, including proteins, lipids, carbohydrates, and nucleic acids. In addition to innate immune cells (of myeloid and lymphoid origin), PRR are expressed by a wide variety of cell types, including endothelial cells, epithelial cells, and adaptive immune cells. Most PRR are involved in the initiation of inflammation and those expressed by professional antigen-presenting cells are critical in initiating antigen-specific immune responses.

PRR can be associated to cells (expressed at the surface or localized intracellularly) or released in the extracellular milieu (sPRM). According to their localization, cell-associated PRR can be classified in three groups (3): (i) membrane receptors, including membrane Toll-like receptors (TLR1, TLR2, TLR4, TLR5, and TLR6), scavenger receptors and C-type lectin receptors, (ii) endosomal receptors, including TLR3, TLR7, TLR8, and TLR9, and (iii) cytoplasmic receptors, including nucleotide-binding oligomerization domain (NOD)-like receptors (NLR), retinoic acid-inducible gene I (RIGI)-like receptors (RLR), and AIM2-like receptors (ALR). Membrane PRR are involved in the clearance of danger signals (i.e., phagocytosis of particulate motifs) and/or cell activation, while intracellular PRR are mainly involved in the detection of nucleic acids.

Soluble PRM act as bridging molecules, linking extracellular DAMPs and PAMPs with cell associated PRR. They include collectins, ficolins, some complement family proteins, soluble forms of membrane PRR (such as soluble scavenger receptors and C-type lectins released after shedding of the membrane forms), and by pentraxins. As their membrane counterparts, sPRM are highly conserved and bind a variety of ligands expressed by microbes and altered/modified self. Soluble PRM recognize the same molecular motifs that cell-associated PRR. They have a role in agglutination, complement activation and opsonisation (i.e., facilitating the recognition and elimination of danger motifs by phagocytes). By reference to their bridging activity, sPRM are usually viewed as ancestors of antibodies (4) and are sometimes referred to as opsonins. They also play a pivotal role in determining the nature of the immune responses induced by the motifs encountered.

A tolerance breakdown to sPRM, evidenced by the presence of autoantibodies, has been reported in different autoimmune diseases, especially autoantibodies targeting members of the pentraxin family. Such autoantibodies have been particularly reported in systemic lupus erythematosus (SLE) and anti-neutrophil cytoplasmic autoantibody (ANCA)-associated vasculitis. In this review, we present the main characteristics and functions of pentraxins and data on anti-pentraxin autoantibodies. Finally, we raise hypothesis on the potential pathological role(s) of anti-pentraxin autoantibodies.

## The Pentraxin Family

### Structure of Pentraxins

Pentraxins are characterized by a C-terminal “pentraxin domain” of about 200 amino acids, containing a highly conserved HxCxS/TWxS motif (where x represents any amino acid) called the “pentraxin signature” (5, 6). They are organized in a cyclic pentameric structure (4, 7). The pentraxin family is divided into two subfamilies, the short and long pentraxins, depending on the length of their amino acid sequences.

Short pentraxins are characterized by a short N-terminal domain (approximatively 20 amino acids). This subfamily contains the acute phase proteins C-reactive protein (CRP), also known as PTX1, and serum amyloid P component (SAP), also known as PTX2.

Long pentraxins are characterized by a long N-terminal domain (178 amino acids). In addition to the prototypic long pentraxin PTX3, this group contains neuronal pentraxin 1 (NP1), NP2, neuronal pentraxin receptor (NPR) and PTX4 (4, 8). Contrary to PTX3, only few data are available on these other members of the long pentraxin subfamily and will thus not be further mentioned in this review.

### Ligands and Immune Functions of Pentraxins

#### The Short Pentraxins CRP and SAP

The short pentraxins CRP and SAP are assembled as multimers of five or ten 25 kDa subunits of 204 and 205 amino acids, respectively. CRP and SAP are the main acute phase proteins in human and mouse, respectively. They are mainly produced by the liver in response to pro-inflammatory cytokines, such as IL-1 and IL-6. Under inflammatory situations, such as bacterial sepsis, sterile inflammation (such as gout) or massive tissue destruction (a process associated with the release of inflammatory endogenous molecules such as HSP or HMGB1), the circulating levels of CRP can rise up to 1,000-time their basal concentrations in human (3 mg/L) (9).

Short pentraxins recognize a large variety of ligands in a calcium-dependent manner. Of note, the first ligand described for CRP was the C-polysaccharide of *Streptococcus pneumoniae*, hence the name. SAP takes its name from the fact that it is one of the universal component of the amyloid deposits found in amyloidosis and Alzheimer’s disease.

CRP and SAP promote microbe opsonisation, thus facilitating their elimination by phagocytes. The functions of short pentraxins are not limited to bacterial clearance as they can also bind many other ligands such as apoptotic cells, amyloid fibrils (10), and endogenous danger molecules, such as histones and nucleic acids released by damaged cells (11, 12).

Short pentraxins are potent regulators of complement pathways. They can activate the classical complement pathway by linking to the complement factor C1q (13) and the lectin complement pathway by binding to ficolins (14, 15). In contrast, they can inhibit the alternative pathway amplification loop by binding to factor H (16). Short pentraxins can also bind to IgG receptors (FcγR). This binding can either inhibit immune complex-mediated phagocytosis or activate FcγR-mediated phagocytosis and cytokine secretion (17–20).

#### The Prototypic Long Pentraxin PTX3

PTX3 is assembled as multimers of 45 kDa (381 amino acids) subunits and is organized as an octamer with monomers linked by disulfide bounds. Unlike short pentraxins, PTX3 is rapidly neo-synthetized by many cell types (4, 5) upon activation by a diversity of signals, including microbes and microbial moieties and inflammatory cytokines (4, 21). Contrary to short pentraxins, PTX3 is constitutively expressed, with circulating levels of ≈ 200 ng/ml in healthy subjects. The levels of PTX3 are strongly and rapidly increased during inflammatory responses (800–1,000 ng/ml). PTX3 is also preformed in neutrophils and rapidly released upon activation (22, 23).

PTX3 binds to the same ligands that short pentraxins, but in a calcium-independent manner (5). Like short pentraxins, PTX3 opsonizes and facilitates the clearance of numerous bacteria (such as *Pseudomonas aeruginosa* and *Salmonella typhimurium*) (24, 25), virus (such as influenza virus) (26), fungi (such as *Aspergillus fumigatus*) (27), and apoptotic cells (4, 5) by phagocytes. As for CRP and SAP, PTX3 can recruit FcγR to induce phagocytosis in a way similar to IgG (17, 18, 20, 28, 29). PTX3 also modulates the complement cascade through its capacity to bind to proteins of the classical (C1q) and lectin (mannose-binding lectin, ficolins) complement pathways (13, 15). PTX3 thus facilitates the tissue deposition of iC3b, thereby promoting complement receptor 3 (CR3)-driven phagocytosis (28). It also increases the binding of activated complement on microbes initiated by ficolin-1. PTX3 interacts with the complement factor H, the main alternative pathway regulatory protein but without modulating its complement inhibitory activity (30). PTX3 also acts as a nodal point for the assembly of the cumulus oophorus hyaluronan-rich extracellular matrix.

Nevertheless, studies have reported that PTX3 may exhibit a pro-inflammatory activity, underlying its “dual complexity” (31). Indeed, PTX3-deficient mice or mice injected with PTX3 develop less severe phenotypes in various models of sterile inflammation (32–34). Especially, a direct role of PTX3 on endothelial cell functions has been described (i.e., dysfunction and morphological changes in the endothelial layer through a P-selectin/matrix metalloproteinase-1 pathway) (35, 36). Considering its diversity of functions, PTX3 is considered as a pivotal sPRM at the crossroad between innate immunity, inflammation, matrix deposition and female fertility (4).

### Pentraxins, Cell Death, and Immune Responses

The management of dying cell clearance is critical to maintain tolerance and to avoid the initiation of auto-immune responses. Schematically, the dichotomy “tolerance versus auto-immunity” is determined by the type of cell death (apoptosis versus necrosis) and the absence or presence of inflammation (either induced by PAMPs or DAMPs) at the time of dying cell clearance.

Apoptosis is characterized by coordinated (programmed) processes allowing dying cells to be eliminated without release of potentially harmful and inflammatory endogenous DAMPs. The clearance of early apoptotic cells is classically viewed as a dynamic tolerogenic process allowing maintaining or inducing regulatory T cell responses. In contrast, necrosis, which usually occurs following a severe aggression, whether sterile or not, is a passive, accidental cell death process accompanied by the release of intracellular components into the extracellular environment, among which are found potent inflammatory and cytotoxic molecules (such as histones and HMGB1) (37). Importantly, the processing of dying cell-derived antigens in an inflammatory context may lead to the activation/maturation of professional antigen-presenting cells and, ultimately, to the activation of self-reactive T cells.

Phagocytosis of apoptotic cells, also called efferocytosis, is a fine-tuned process, involving at least three molecular partners (38): (i) eat-me molecules (i.e., neo-expressed motifs allowing discriminating live and dying cells), (ii) sensing and internalization receptors expressed by phagocytes, such as complement component receptor (which recognize collectins), scavenger receptors, integrins, and other receptors (e.g., MER and CR3/CR4), and (iii) sPRM acting as bridging molecules between dying cells and phagocytes.

The pivotal role of opsonins in the clearance of apoptotic cells has been illustrated in C1q- and SAP-deficient mice. C1q-deficient mice spontaneously develop signs of autoimmunity (39) and mice with targeted deletion of the SAP gene spontaneously develop antinuclear autoimmunity and severe glomerulonephritis, a phenotype resembling human SLE (40).

While CRP and SAP have a facilitating role (11, 41, 42), the role dedicated to PTX3 remains unclear. Indeed, our team has demonstrated that preformed PTX3 contained within neutrophil granules is relocalized at the surface of dying neutrophils, thereby acting as an “eat-me” molecule to mediate their capture by phagocytes (43). In contrast, a study reported that PTX3 binds selectively to late apoptotic cells and inhibits their capture by phagocytes (42, 44) suggesting that short and long pentraxins, in the extracellular milieu, may have opposite impacts on apoptotic cells clearance.

It remains also difficult to reconcile *in vitro* and *in vivo* data. On the one hand, and contrary to what would have been suspected based on *in vitro* data, SLE-prone mice supplemented with CRP and CRP-transgenic mice develop a mild kidney disease (45–47). On the other hand, and in agreement with *in vitro* data, C57/BL6 SAP-deficient mice can produce autoantibodies and develop immune complex glomerulonephritis (40, 48). It is important to note that the genetic background is important in enabling autoimmune phenotype expression. Indeed, SAP^-/-^ C57/BL6 but not SAP^-/-^ 129/Sv mice, spontaneously develop autoantibodies (48). Moreover, the authors also reported that SAP^-/-^ and SAP^-/-^ human SAP transgenic mice exhibit a similar autoimmune profile, suggesting that differences may exist between the human and mouse SAP in their capacity to interact with DAMPs. A similar complexity has been reported in C1q-deficient mice, as C1q^-/-^ C57/BL6×129Sv but not C1q^-/-^ C57/BL6 mice spontaneously develop autoimmunity and glomerulonephritis (39). These studies underline the important of genetic background of mice in their susceptibility to develop autoimmunity, suggesting that tolerance breakdown could be strain dependent or dependent on the strategy to knock out target gene expression (impact of genetic recombination on the expression of other key genes) and/or that differences exist between human and mouse sPRM in their capacity to interact with DAMPs. To the best of our knowledge, PTX3-deficient mice do not spontaneously develop signs of autoimmunity. However, they develop a more severe phenotype in a ischemia reperfusion injury model (49) but exhibit a reduced inflammation in an acute arthritis model (50), even though both models are associated with a strong inflammation and, certainly, consecutive or associated to cell death. Moreover, PTX3-deficient mice bred with SLE prone mice have a decreased ability to clear apoptotic cells and tissue damages are aggravated in lung but not in kidney. Collectively, these results suggest that PTX3 deficiency may favor tissue injury associated to the initiation of an autoimmune response (51).

The potential role of pentraxins during auto-immune diseases has been reinforced by translational studies. CRP has been found decreased during SLE flares whereas inflammation markers were elevated (52, 53), suggesting that low levels of CRP may be associated with an impaired efferocytosis (54). Moreover, we reported that the levels of PTX3 are lower in patients with SLE during active disease as compared to healthy subjects (55). Whether these decreases impact the efferocytosis process remains undetermined. Nevertheless, these results suggest that a decrease of pentraxin levels which may lead to a reduced efferocytosis, is associated with an autoimmune signature that can be induced by the processing of dying cells in an inflammatory environment.

Collectively, and although the predominant roles of pentraxins seem to be host-protective, detrimental effects were observed in certain experimental settings. This so-called “yin-yang” effect may be inherent to the multi-functional properties of pentraxins, the complexity of efferocytosis, the redundancy between sPRM and the potential interaction of pentraxins with different DAMPs released by dying cells (56).

## Anti-Pentraxin Antibodies in Autoimmune Diseases

Antibodies directed against CRP, SAP, and PTX3 have been detected in various autoimmune diseases, especially in SLE and ANCA-associated vasculitis (AAV). Their potential roles in the pathogenesis of autoimmune diseases are described thereafter.

### Anti-Pentraxin Autoantibodies in SLE

SLE is an autoimmune disease with a very heterogeneous clinical presentation. The prognosis is mainly dependent on renal impairment, affecting 50% to 70% of patients. SLE is characterized by the production of autoantibodies directed against a large panel of self-antigens, including mainly nuclear antigens (DNA, soluble nuclear antigens (Sm), ribonucleoproteins (RNP) and histones) but also anti-pentraxin Abs. SLE is also characterized by the activation of complement and the deposition of immune complexes that lead to systemic tissue damages (especially renal, vascular, hematological, cutaneous, and articular lesions) (57). The emergence of autoantibodies is determined by environmental, hormonal, and genetic factors suspected to be involved in the dysregulation of the innate and adaptive immune systems. In a mechanistic point of view, SLE is driven by a strong activation of dendritic cells and the production of type I interferons and is associated to an altered efferocytosis (57, 58).

#### Anti-CRP Antibodies in SLE

Autoantibodies directed against CRP bind to the monomeric form but not the pentameric form (59). The dissociation of a pentameric to a monomeric form of CRP has been reported in inflammatory situations (60) and monomeric CRP accumulates at the surface of various cell types (B cells, NK cells, platelets) and in inflamed tissues (61).

Anti-CRP Abs have been initially reported in SLE (**Table 1**) (76). They are present in a significant proportion of patients, ranging from 22% to 78%. Only one study (65) did not found an increased prevalence of anti-CRP Abs in SLE patients compared to healthy subjects. This conflicting result may be due to different methods of Abs determination.

**Table 1:** Prevalence of anti-pentraxin antibodies in different autoimmune diseases.

HS	SLE	PAPS	SSc	SS	RA	AAV	Reference
**Anti-CRP Abs, n (%)**							
1/40 (2.5%)	39/50 (78%)	–	2/20 (10%)	–	–	–	(59)
5/100 (5%)	13/27 (48%)	–	–	2/16 (12.5%)	0/15 (0%)	–	(62)
4/80 (5%)	77/137 (51%)	68/127 (54%)	–	–	–	–	(63)
–	43/190 (22.6%)	–	–	–	23/103 (22.3%)	–	(64)
2/45 (4.4%)	12/150 (8%)	–	–	–	–	–	(65)
0/60 (0%)	20/49 (40.8%)* 56/96 (59.3%)**	–	–	–	–	–	(66)
1/34 (2.9%)	18/81 (22.2%)	–	–	–	–	–	(67)
0/60 (0%)	–	–	–	2/7 (28.6%)	–	1/20 (5%)	(68)
–	26/100 (26%)	–	–	–	–	–	(69)
21.01 ± 14.32	35.6 ± 35.1	–	–	–	–	–	(70)
5/92 (5.4%)	–	–	–	–	–	11/120 (9.2%)	(71)
10/36 (27.8%)	18/34 (52.9%)	–	–	–	–	–	(72)
**Anti-SAP Abs, n (%)**							
3/124 (2.4%)	145/328 (44%)	–	–	–	–	–	(73)
1/45 (2.2%)	20/100 (20%)	–	–	–	–	–	(65)
3/93 (3.2%)	–	–	–	–	–	21/120 (17.5%)	(71)
**Anti-PTX3 Abs, n (%)**							
4/93 (4.3%)	18/36 (50%)	–	–	–	1/40 (2.5%)	–	(55)
8/130 (6.2%)	60/130 (46.2%)	–	1/26 (3.8%)	2/26 (7.7%)	2/27 (7.1%)	–	(74)
12/227 (5.3%)	–	–	–	–	–	56/150 (37.3%)	(71)
2/100 (2%)	38/196 (19.4%)** 61/150 (40.7%)*	–	–	–	–	–	(75)

HS, healthy subjects; SLE, systemic lupus erythematosus; PAPS, primary antiphospholipid syndrome; SSc, systemic sclerosis; SS, Sjögren syndrome; RA, rheumatoid arthritis; AAV, ANCA-associated vasculitis.

^∗^SLE patients without lupus nephritis.

^∗∗^SLE patients with lupus nephritis.

The correlation between the presence of anti-CRP Abs and clinical presentation at disease onset was not systematically evaluated (59, 62). In a study including 137 patients with SLE, Figueredo et al. showed that patients with anti-CRP Abs also had elevated levels of anti-double strand (ds) DNA and anti-phospholipids Abs and low levels of the complement component C3. The frequency of anti-CRP Abs was also more frequent in patients with nephritis (27% and 13% in patients with or without nephritis, respectively) (63). Nevertheless, this result was not confirmed in another study (69). In patients with nephritis, anti-CRP Abs were also more frequent (66). Moreover, patients with anti-CRP Abs also had a higher SLEDAI score (66, 70) and were more likely to present with acute renal injury (66). However, these results must be nuanced as no relation between disease activity and presence of anti-CRP Abs was found by others (67, 72). Focusing on renal involvement, a significant correlation was found between anti-CRP Abs and acute renal tubulointerstitial injury (inflammation and fibrosis) but not with specific glomerular lesions (66). Collectively, these data suggest that anti-CRP Abs reflect SLE activity rather than a specific pattern of SLE nephritis.

#### Anti-SAP Antibodies in SLE

To the best of our knowledge, only two studies reported the prevalence of anti-SAP Abs in SLE (65, 73) (**Table 1**). In two studies performed by the same team, Zandman et al *(* 73 *).* included 328 patients with SLE and found anti-SAP Abs in 44% of patients, slightly above the 20% found in the second study, as compared with 2% in healthy subjects (65). As for anti-CRP Abs, anti-SAP Abs were not associated with a specific disease phenotype (73), despite an association with anti-dsDNA titers and disease severity index. The levels of auto-Abs lowered under treatment.

#### Anti-PTX3 Antibodies in SLE

Anti-PTX3 autoantibodies were first described in SLE patients by our team (55). Their strong prevalence (≃ 50% of patients, as compared to 4% in healthy subjects) was later confirmed in an independent study (74). In our cohort, we observed an association between anti-PTX3 Ab levels and disease activity (assessed by the SLEDAI score). Antibodies titers were also correlated with classical SLE activity biomarkers, such as anti-dsDNA Abs, anti-nuclear Abs and C3/C4 complement components levels (55). However, the study by Bassi et al. did not found any association between anti-PTX3 antibody positivity and SLE activity (74). The authors assessed disease activity using the ECLAM (European Consensus Lupus Activity Measurement) score, making it difficult to compare the two studies. Interestingly, in this study, the authors suggested that anti-PTX3 autoantibodies might provide protection from renal involvement as they represent an independent factor negatively associated with kidney injury. Similar results were reported in a recent study evaluating the prevalence of anti-PTX3 Abs in SLE (75). In this large cohort study, including 246 SLE patients and 100 healthy subjects, Yuan et al. found that patients with SLE nephritis had a lower prevalence of anti-PTX3 Abs compared with patients without nephritis (20% versus 40%, respectively). Moreover, the serum levels of anti-PTX3 Abs were negatively correlated with proteinuria in lupus nephritis. The levels of proteinuria, serum creatinine and the prevalence of thrombotic microangiopathy were also higher in patients without anti-PTX3 Abs. Although it did not reach statistical significance, patients with anti-PTX3 antibodies were less likely to reach end stage kidney disease. PTX3 deposition and renal fibrosis were also significantly lower in anti-PTX3 Ab-positive patients than negative patients (74, 77). These results are consistent with data in animal models. Indeed, lupus prone NZB/NZW mice immunized with PTX3 produce anti-PTX3 Abs and have a delayed occurrence of nephritogenic Abs, a decreased proteinuria, and an increased survival (78).

These results suggest that the balance between the levels of serum PTX3 and anti-PTX3 autoantibodies might influence the nephritic process (progression or attenuation). Anti-PTX3 Abs are not only markers of the inflammatory status in SLE but may also be protective by counteracting the deleterious effects of tissue deposition of PTX3 or complement activation, especially in kidneys. In support, the occurrence of anti-dsDNA and anti-C1q Abs in PTX3-immunized mice was significantly delayed and their levels increased when anti-PTX3 autoantibodies were decreasing. Hence, anti-PTX3 antibodies may prevent the proinflammatory deposition of PTX3 in target organs, decreasing the amount of available autoantigens in inflamed kidneys. This suggests that anti-PTX3 Abs might inhibit the secretion of nephritogenic Abs in lupus nephritis (78).

### Anti-Pentraxin Antibodies in AAV

ANCA-associated vasculitis (AAV) include granulomatosis with polyangiitis (GPA), micropolyangiitis (MPA) and granulomatosis with polyangiitis and hypereosinophilia (EGPA). AAV are systemic pathologies related to small vessel inflammation whose prognosis is mainly linked to renal (rapidly progressive glomerulonephritis) and pulmonary (intra-alveolar hemorrhage) damages (79). These conditions can rapidly become life-threatening or lead to irreversible functional impairment of the affected organ if they are not promptly treated. Renal or pulmonary impairment can be associated with skin, ear-nose-throat, joint, or neurological damages. These autoimmune diseases are frequently associated with the presence of ANCA, which are diagnostic markers in 80%–90% of patients (80). ANCA-negative vasculitis have been described, especially in patients with EGPA (up to 50%) but also in patients with GPA or MPA (up to 10%) (81–83), raising the possibility of other autoantibodies which remain to be identified. The identification of these autoantibodies should be useful for the formal diagnosis of AAV, as early diagnosis is essential to initiate appropriate treatment in order to limit irreversible organ injuries and favor long-term patient survival (84). Some ANCA targeting “minor antigens” have been described (directed against bactericidal/permeability increasing protein, elastase, and cathepsin G) in various autoimmune diseases, especially connective tissue diseases, but their pathogenic role in AAV remains to be demonstrated (85, 86). Antibodies against pentraxins have been described in AAV patients.

#### Anti-CRP Antibodies in AAV

As reported by our group and by Tan et al. (68, 71), anti-CRP Abs were found more frequently in AAV patients as compared to healthy controls, but the levels were similar levels in both groups. To our knowledge, no other study addressed this issue.

#### Anti-SAP Antibodies in AAV

In our study, the only one to our knowledge that evaluated the prevalence of anti-SAP Abs in AAV patients (71), anti-SAP Ab titers were higher in AAV patients than in healthy subjects. Moreover, 17.5% of AAV patients were positive for anti-SAP Abs compared with 3.2% in the control groups. Among the anti-SAP-positive patients, two were ANCA negative, suggesting that anti-SAP Abs could represent a diagnostic marker in this group of patients.

#### Anti-PTX3 Antibodies in AAV

The fact that PTX3 is constitutively expressed by fully differentiated (mature) neutrophils suggests that it may represent a potential target for ANCA. Unlike short pentraxins which are synthetized in the liver (9), PTX3 is stored into neutrophil-specific granules (87). Moreover, PTX3 can be localized in neutrophil extra-cellular traps (NETs) with other intracellular proteins targeted by these autoantibodies, such as MPO and PR3 (56, 88). NETs result from a particular type of neutrophil death called NETosis (89), which is characterized by the release of the intracellular content, including genomic DNA. Extracellular DNA thus forms a network to which are associated intracellular molecules such as PR3 and MPO. NETs are microbicide, contributing to fight extracellular infectious agents. However, NETs can also be cytotoxic for endothelial cells and tissues during sterile inflammation. NETosis favors tolerance breakdown, promoting autoimmunity as evidenced by the generation of ANCA through dendritic cell-mediated antigen presentation (90, 91). Moreover, ANCA can induce NETs, which can, in turn, activate the complement alternative pathway (92–94), leading to an amplification loop of neutrophil activation and death. Indeed, C5a will further attracts and primes neutrophils, rendering them more sensitive to ANCA-mediated activation.

Anti-PTX3 Abs were assessed in the serums of 150 AAV patients by our team (71). Nearly 40% of AAV patients had anti-PTX3 Abs. Moreover, Abs titers were higher in patients with active than in those with inactive AAV. In our cohort, 14 patients (6 EGPA, 6 MPA, and 2 GPA) were positive for indirect immunofluorescence (IIF) ANCA screening, but negative for major (MPO, PR3) and minor (lactoferrin, BPI, elastase, and cathepsin G) ANCA antigens. Interestingly half of them (7/14) had detectable anti-PTX3 Abs. Moreover, anti-PTX3 Abs were found to have a unique IIF pattern known as “small cytoplasmic” ANCA characterized by small-size cytoplasmic granules in methanol- and ethanol-fixed neutrophils, and less intense in formol-fixed neutrophils.

Nevertheless, we did not find any clue for a pathological role of these Abs. Indeed, no difference of renal outcome between anti-PTX3 autoantibody-positive and -negative patients was observed and no relation between anti-PTX3 Ab levels and kidney function at the time of AAV diagnosis was observed (personal unpublished data). It would probably be interesting to evaluate the prevalence of anti-PTX3 Abs in a larger cohort of ANCA-negative patients before to conclude on their clinical relevance and predictive value.

We may wonder whether anti-PTX3 Abs are really ANCA. Indeed, anti-PTX3 Abs were found positive by immunofluorescence on fixed neutrophils and anti-PTX3 Ab positivity was observed in ANCA-negative AAV patients. Finally, the biological role(s) of anti-PTX3 Abs remain(s) to be evaluated. The purification of anti-PTX3 Abs from the serum of patients without other detectable ANCA would allow analyzing the *in vitro* biological activity of these autoantibodies.

### Anti-Pentraxin Antibodies in Other Autoimmune Systemic Diseases

#### Anti-Short Pentraxin Antibodies

Anti-CRP Abs have been identified in other autoimmune diseases, such as systemic sclerosis (SSc), rheumatoid arthritis (RA), and Sjögren syndrome (SS) patients, although at a lower prevalence than in SLE patients (**Table 1**). Interestingly, a high prevalence of anti-CRP Abs (54%) has been reported in patients with primary anti-phospholipid syndrome (PAPS) (63). Patients with PAPS and anti-CRP Abs were more likely to present thrombosis or fetal loss (55% vs 20%). More studies are needed to evaluate if anti-CRP Abs could be used as a biomarker for thrombosis risk assessment.

To the best of our knowledge, the presence of anti-SAP Abs in disease other that SLE or AAV has not been reported.

#### Anti-PTX3 Antibodies

Anti-PTX3 Abs have been detected in other autoimmune diseases such as SSc, SS and RA, but at a frequency similar to healthy subjects (74).

## Anti-Pentraxin Antibodies: Pathogenic Actors or Bystander Markers?

The presence of anti-pentraxin autoantibodies has been reported mainly in SLE and AAV. Interestingly, antibody levels are generally high at disease onset and decline during remission phases, suggesting that they may represent, at least, diagnostic markers.

It should be noted that, given the strong homology between pentraxins, and in particular between the short pentraxins CRP and SAP, the potential cross-reactivity of autoantibodies directed against pentraxins can be a source of error on the nature of the target as well as on their potential pathological roles.

At this time, we do not know whether these antibodies reflect only tolerance breakdown, like many other autoantibodies reported in these pathologies, or whether they play a pathophysiological role. A potential pathological role for autoantibodies is supported by data on MPO-ANCA for which a pathogenic role is highly suspected. In addition to their capacity to activate neutrophils, MPO-ANCA induce glomerulonephritis when injected in mice (95) and vasculitis in newborns after placental transmission (96). Although neonatal lupus with congenital atrioventricular block after placental transmission anti-Sjögren’s-syndrome-related antigen A (SSA) Abs has been described (97), the pathogenicity of antinuclear Abs remains a matter of debate.

To date, no experimental *in vitro* or *in vivo* data supports a pathogenic role for anti-pentraxin Abs. Purifying anti-pentraxin Abs may allow to evaluate their potential pathological role, as previously described for anti-MPO and anti-PR3 Abs. However, the cross-reactivity of anti-pentraxin antibodies, which is more than likely due to strong sequence (especially between CRP, SAP, and the C-terminal domain of PTX3) and structure homologies (accessibility to shared conformational epitopes exposed at the surface of pentraxins), makes it difficult to draw a conclusion. Moreover, different epitopes of each pentraxin could be recognized by different antibodies, and the resulting functional outcomes may have different consequences. The isotype of the anti-pentraxin autoantibodies, the site of production (tissue versus mucosal barriers) and the immune status of the tissue at the time of efferocytosis (such as chronic inflammation) can also have important consequences on the regulatory versus activating potential of these autoantibodies. Such complexity could lead to misinterpretation of the roles of anti-PTX Abs in the pathogenesis of autoimmune diseases.

Nevertheless, and based on the role of pentraxins in efferocytosis, it is tempting to speculate that these autoantibodies could alter the efferocytosis process and, consequently, the outcome of immune responses against self-antigens.

**Figure 1:**
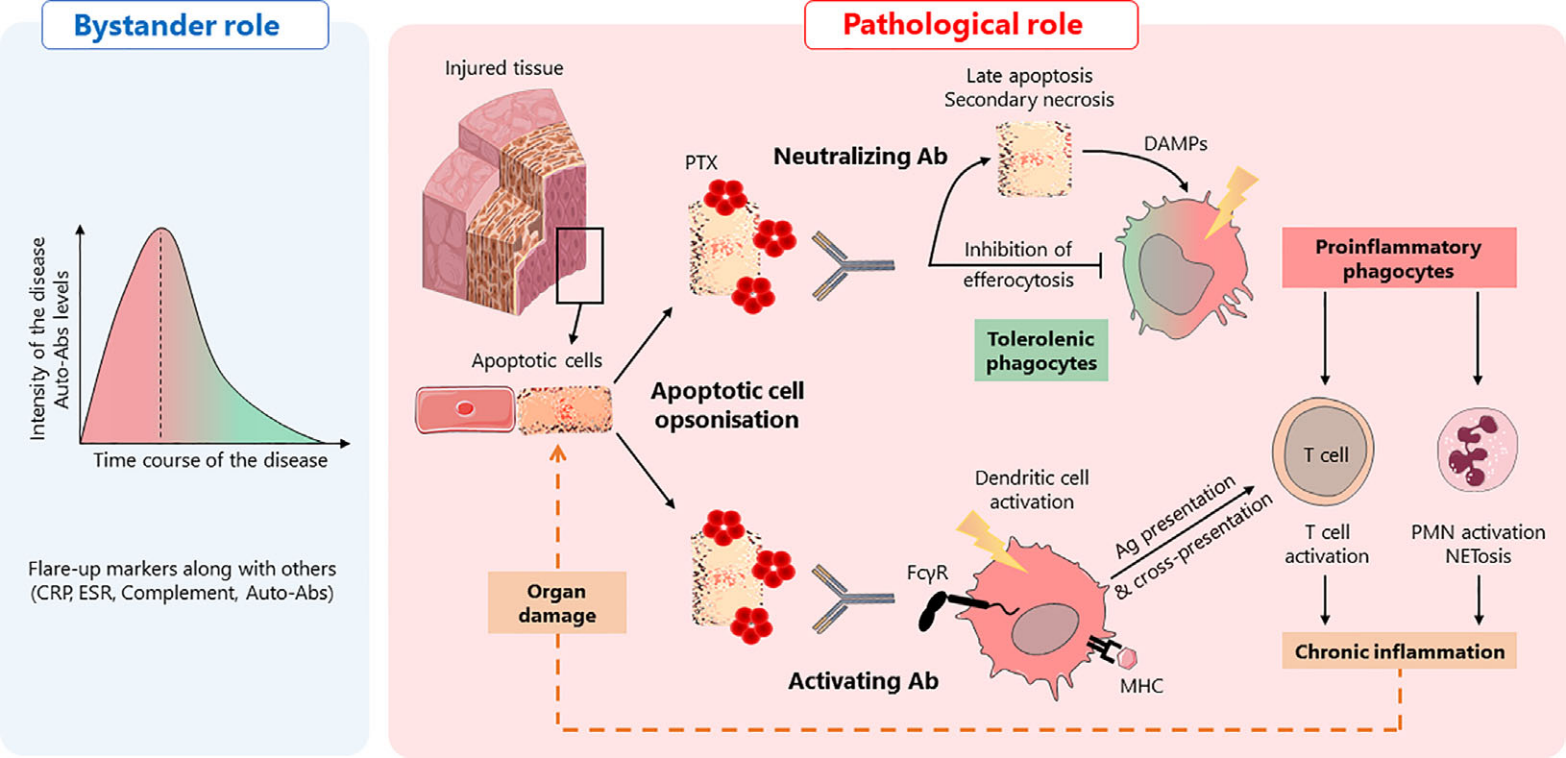
Anti-pentraxin antibodies in autoimmunity: a hypothetical model. Anti-pentraxin antibodies are found elevated at autoimmune disease onset and decline during remission phases, suggesting that they are, at least, diagnostic markers, playing a **“**bystander role**”** (left panel). Based on the role of pentraxins in efferocytosis, two hypotheses can be drawn about a hypothetical **“**pathological role**”** of anti-pentraxin Abs (right panel). In a physiological context, apoptotic cells are opsonized by pentraxins (PTX) that facilitate their engulfment. This process promotes tolerogenic phagocytes and prevents inflammation. The presence of anti-pentraxin Abs can lead to the initiation of adaptive immune responses against dying cell antigens and NETosis, a pro-inflammatory process associated with the release of autoantigens. First, anti-PTX Ab may behave as neutralizing antibodies, inhibiting apoptotic cells engulfment by professional antigen-presenting cells, leading to the evolution of dying cells from early to late apoptotic/necrotic cells. These dying cells release potent inflammatory DAMPs inducing the activation of antigen-presenting cells. Second, anti-PTX Ab can be considered as activating Ab, inducing the phagocytosis of dying cells *via* FcγR. This process also induces the activation of antigen-presenting cells, favoring the initiation of MHC-I and MHC-II immune responses through antigen presentation and cross-presentation. Whatever the **“**function**”** of anti-pentraxin Ab, they initiate an inflammatory process leading to a deleterious amplification loop responsible for tissue damages.

On the one hand, anti-pentraxin Abs could inhibit or delay apoptotic cells engulfment by professional antigen-presenting cells, leading to the evolution of dying cells from early to late apoptotic cells. Importantly, late apoptotic/secondary necrotic cells can release potent inflammatory DAMPs that can activate antigen-presenting cells and, consequently, induce the initiation of humoral and cellular specific immune responses. On the other hand, anti-pentraxin Abs may induce the phagocytosis of PTX-opsonized dying cells *via* FcγR, a process that favors antigen-presenting cell activation and antigen processing that, ultimately, favors the initiation of MHC-I and MHC-II immune responses (98, 99). Moreover, while the interaction of apoptotic cells with pentraxins usually has an opsonizing role, a reduced internalization of apoptotic cells by dendritic cells has been reported for PTX3. The authors described this mechanism as “PTX3 censorship”, i.e., masking of “eat-me” signals rendering them not detectable by phagocytes (100). Antibodies directed against PTX3 could therefore re-confer immunogenicity to apoptotic cells by promoting the presentation of apoptotic cell-associated antigens by professional APCs and, consequently, the priming and/or expansion of self-reactive T cells. Even though speculative, both mechanisms, which are not exclusive and could occur concomitantly, may favor/maintain the development of autoimmune responses directed against dying cell-associated antigens (**Figure 1**). Additional studies need to be performed to elucidate the complex dialog between soluble and membrane associated PTX3 (soluble versus) with anti-PTX3 Abs and consequences on sensing and processing of apoptotic cells. Finally, a lower frequency of anti-PTX3 Abs have been reported in patients with lupus nephritis, suggesting that these autoantibodies could also be protecting against PTX3 or complement deposition in kidneys, further enhancing the complexity of the potential pathogenic role of anti-pentraxin auto-antibodies.

Nevertheless, we cannot exclude a protective role for anti-pentraxin Ab, notably in kidney disorders associated with anti-pentraxin Abs. Indeed, anti-pentraxin Abs could inhibit the pentraxin deposition in tissue, thereby preventing complement binding and activation. Whether this process may occur *in vivo* remains undetermined. Another original mechanism has been suggested for anti-SAP Abs. Bickerstaff M et al have reported that SAP inhibits the generation of pathogenic anti-DNA autoantibodies, probably by delaying chromatin degradation (40). This process could be inhibited by anti-SAP Abs, thereby favoring or amplifying the generation of autoantibodies and inflammation.

Nevertheless, it is important to underline that these processes are based on the hypothesis that anti-pentraxin antibodies exhibit mostly similar biological properties. These scenarios can be very different, both in terms of magnitude and nature of the immune response induced, depending on the diversity of autoantibodies produced (isotype, levels) and the presence or absence of each of these pentraxins. The scenario may be even more complicated, particularly due to the involvement of other sPRM and PRR involved in the recognition and removal of danger signals.

## Concluding Remarks

Pentraxins are soluble PRM involved in antimicrobial defense and efferocytosis. A tolerance breakdown to pentraxins has been evidenced during various autoimmune diseases. Especially, anti-PTX3 autoantibodies are frequent in SLE and AAV and may be of interest for diagnosis, disease activity, response to treatment and outcomes. Moreover, anti-PTX3 Abs share some characteristics with ANCAs and are associated with a specific indirect immunofluorescence neutrophil staining, suggesting that they differ from other ANCA. The prevalence of anti-PTX3 Abs appears to be high during ANCA-negative AAV and could constitute a useful marker in these settings. Prospective longitudinal clinical studies are required to confirm the potential of anti-PTX3 Abs as diagnosis and prognosis markers. In a mechanistic point of view, studies are required to determine the potential pathogenic role of anti-PTX3 and whether they should be targeted if they are more than just bystanders in severe autoimmune diseases.

## Author Contributions

BB wrote the first draft of the manuscript. YD and J-FA provided critical revision of the manuscript. All authors contributed to the article and approved the submitted version.

## Funding

This study did not receive any specific grant from funding agencies in the public, commercial, or not-for-profit sectors. This work was realized in the context of (i) the LabEX IGO program (National Research Agency; ANR-11-LABX-0016-01) and (ii) the University Hospital of Angers - University of Angers joint program (project 3I-Impact).

## Conflict of Interest

The authors declare that the research was conducted in the absence of any commercial or financial relationships that could be construed as a potential conflict of interest.

## Abbreviations

AAV, ANCA-associated vasculitis; Ab, antibody; ANCA, anti-neutrophil cytoplasm antibody; CRP, C-reactive protein; DNA, deoxyribonucleic acid; MHC, major histocompatibility complex; MPO, myeloperoxidase; PAPS, primary anti-phospholipid syndrome; PR3, proteinase 3; PTX, pentraxin; RA, rheumatoid arthritis; SAP, serum amyloid P; SLE, systemic lupus erythematosus; SLEDAI, SLE activity index; SS, Sjögren syndrome; SSA, Sjögren’s-syndrome-related antigen A; SSc, systemic sclerosis.
